# On Certain Conditions of the Dental Tissues

**Published:** 1856-07

**Authors:** John Tomes

**Affiliations:** Surgeon-Dentist to the Middlesex Hospital.


### SELECTED ARTICLES.

### ARTICLE VIII.

*On Certain Conditions of the Dental Tissues.* By John Tomes, F. R. S., Surgeon-Dentist to the Middlesex Hospital.

The temporary teeth, when about to be replaced by the permanent set, lose their fangs by gradual absorption of their substance. The crown, when thus left, having but little hold upon the gum, soon falls out. The manner in which the absorption of the dental tissues is effected has been described in a paper published in the "Philosophical Transactions," in 1853. The subject is there mentioned in connection with the absorption of bone.

Having latterly had occasion to devote considerable attention to the phenomena attending the casting off of the deciduous teeth, several conditions relative to absorption have come under my notice, which, as applied to teeth, had, I think, hitherto escaped observation. It may, however, be here stated, that the more recent examinations have not led to any modification of the opinions upon the subject of absorption advanced in the paper alluded to, but have served rather to confirm the statement there made. Absorption may commence upon any part of the fangs of a tooth, and at several points at the same time. By the gradual extension of this process, both in depth and superficially, the root of the tooth is wasted, till, at last, nothing is left but the crown, and even this part is often so much hollowed out, that, excepting the enamel, but little of the tooth remains. The *cementum* is first attacked, then the *dentine* disappears, and the enamel at those points where the *dentine* has been entirely removed suffers from the same action. But whichever of the three tissues is attacked, we see the same characteristic surface as that shown by bone when undergoing a similar action, namely, a surface full of deep indentations, as though they had been made by a sharp piercing instrument, having a semicircular extremity. These minute holes or depressions proceed in various directions, several advancing from contrary points towards the same spot, not unfrequently isolate pieces of *dentine.* If a section be taken through the substance of a tooth, so as to cut the wasting part at a right angle, we shall find the surface acted upon to have an irregular festooned outline, so characteristic, that when once seen it cannot fail to be again recognized.

It has been stated that, closely applied to the surface, a cellular mass will be found, and that this is but slightly adherent, the wasting and growing surfaces readily parting, unless the two are held together by the irregularities on the surface of the former. It will sometimes happen that the cellular mass penetrates into the *dentine* through a small opening, and there dilates, in which case its withdrawal becomes impossible. This condition is now and then found on sections prepared for the microscope, when we have an opportunity of examining the two tissues *in situ.* Indeed we shall find a few cells adherent to the surface of the *dentine* where less deep burrowing has occurred. The cells themselves do not present any peculiarity by which they could be readily recognized, if separated from the part undergoing removal. They are small granular cells, of a more or less spherical form. If a tooth which has lost its fang be carefully removed, we shall find remaining in its place a growing *papilla,* corresponding exactly in size and form to the surface from which it has been separated; and this separation may often be effected with so little injury to the absorbent organ, that no blood appears upon its surface after the operation, although the organ is highly vascular and readily torn.* The superficial extent of the papilla will be equal to that part of the tooth undergoing waste, but the extent, as regards depth, is slight, for, as the root of the tooth disappears, the socket is contracted by the deposition of bone, which forms at the base of the absorbent organ as rapidly as the cellular surface encroaches upon the tooth. The cases in which we find an exception to this condition are those in which the permanent has advanced close to the fangs of the temporary tooth, when the crypt containing the one communicates with the socket of the other, the rate of growth of the permanent having been greater than the absorption of the deciduous organ; but even in these cases we may generally observe some part in which the contraction of the socket is coincident with the absorption of the occupant fang. From the following quotation, it does not appear that Mr. Bell observed these conditions:—

"It has been already stated, that the permanent teeth during their formation are crowded together in the jaw, by being placed in a smaller arch than they would occupy if regularly placed side by side. As the latter, however, is their destined situation, we find that as soon as they are advanced to a certain point of their formation, and can no longer be contained within the *alveoli*, absorption takes place in the anterior parietes of the cavities, by which means the teeth are allowed to come in some measure forward. In consequence of this absorption it often happens, that not only the socket of the corresponding temporary tooth, but that of the tooth on each side is also opened to the permanent one. Absorption now commences in the root of the temporary tooth, generally on that part nearest its successor, and thus goes on by degrees as the latter advances, until the root is completely removed, the crown at length falls off, leaving room for the permanent tooth to supply its place."

Mr. Bell, however, rejects the idea that mere pressure of the one tooth against the other has any thing to do with the absorption of the first set; an opinion that he would probably have expressed even more strongly, had he observed the shallow but perfect sockets which are formed when the temporary teeth are shed before their successors are ready to appear. This, however, must be a very common

* Laforgue and Bourdet recognized the presence of the absorbent organ, but supposed it exhaled a fluid capable of dissolving the roots of the temporary tooth.

condition, as I have in my own collection several specimens illustrating the point.

The fact was not overlooked, I think, by Hunter, although his description is not very clear. He states at page 99 in his 'Natural History of the Teeth,' "The new *alveoli* rise with the new teeth, and the old *alveoli* decay in proportion as the old teeth decay; and when the first set falls out, the succeeding teeth are so far from having destroyed, by their pressure, the parts against which they might be supposed to push, that they are still inclosed and covered by a complete bony socket. From this we see that the change is not produced by a mechanical pressure, but by a particular process in the animal economy."

But there is still a disposition on the part of many who are intrusted with the treatment of teeth, to attribute the absorption of the roots of the one tooth to pressure occasioned by the growth of its successor, and the development of the permanent may have something to do with the shedding of the other. But this does not offer a satisfactory explanation of all the circumstances attending the absorption of the fangs of teeth. In the first place we sometimes meet with cases in which the fangs of permanent teeth are as completely absorbed as those of the temporary organs. Then, again, the fangs of temporary teeth, which have no successors, are also absorbed. These circumstances, taken with the hitherto overlooked fact, that with the waste of the temporary tooth we have pretty generally a corresponding development of bone within the socket to be removed before the permanent tooth appears through the gum, render the pressure theory somewhat unsatisfactory. Another condition may be adduced, tending also against that opinion, namely, that temporary teeth occasionally maintain their place to the exclusion of the permanent ones, which are then kept within the substance of the jaw, or appear in some unusual position.

The relation as regards time between the absorption and shedding of temporary teeth and the appearance of the succeeding permanent teeth are by no means constant. In some cases the temporary teeth are thrown off two years before the corresponding permanent ones come through the gums. In others, again, the new will replace the old ones in as many weeks or even days.

Before the laws which regulate the absorption of the fangs of teeth can be fully recognized, a more perfect knowledge of the condition attending the process must be acquired. Recent examinations have enabled me to add the following additional facts bearing upon this subject to those already known. The process of absorption once commenced, it appears to have been assumed that the same action would be continued, with more or less rapidity, until the tooth falls out; or if not continual, is suspended only. Such, however, is not constantly the case. Not only is the action of absorption suspended, but one of development takes its place. We find the excavated surface of the *dentine cementum* and enamel covered with *cementum*, the latter following all the irregularities of the former tissues, and closely united to them. In cases where this development is going on, or being set up is maintained, the teeth afford considerable resistance when their removal is attempted. In those instances where the first teeth have remained, and tend to the displacement of the second set, this deposit of *cementum* will be found to exist in considerable quantity.

The development of bone upon the surface which had formerly been the seat of absorption, by no means indicates that the tooth will not again be subject to destructive action. On the contrary, specimens in my collection show that the bone deposited under the above circumstances may itself become the subject of absorption, that this process may be again suspended and development be renewed, that the absorption may again take the place of development; in fact, that wasting and reparation may alternate until by the preponderance of the former the tooth is shed. In sections of teeth showing this peculiar condition of development, we may find upon the growing bone numerous osteal cells, with here and there a lacunal cell. A bone *lacuna*, situated within a semicircular indentation in the *dentine*, gives the appearance of a lacunal cell, and a *lacuna* similarly situated in the *cementum* (a circumstance of common occurrence,) has possibly been supposed by Mr. J. Salter to be what has been described in the paper before referred to as a lacunal cell.*

The part of a tooth which has the greatest power of resisting absorption, is that in immediate contact with the pulp. We find examples in which a thin shell of *dentine* surrounds that organ, while that around it has been in great part taken away. This is, however, eventually removed, and the pulp itself changes its character, and becomes an absorbent organ, or makes way for that which is. In a fortunate selection we may find sections showing in one part *dentine* which has been but recently formed, with its modular outline and contiguous cells, capable of developing *dentine;* in another part absorption in active progress; and in a third the deposition of bone on the surface of the wasted *dentine.* In no instance, however, have I seen *dentine* deposited upon the surface of that which has been diminished by absorption.

It would appear that the dentinal pulp, although its function may be changed into that of absorption, or its place be taken by an absorbent organ, and this, again, changed to one for the development of bone, is incapable of resuming under any recognized circumstances its primary function of dentinal development. In other words, that a portion of *dentine* when removed by absorption, cannot be replaced;†

* Transactions of the Pathological Society, vol. vi, p. 169.

† Since the manuscript was sent to the Editors of this Journal, I have seen a paper published in the last number of the Guy's Hospital Reports, by Mr. J. Salter, 'On Intrinsic Calcification of the Permanent Tooth-pulp.' Mr. Salter describes a section taken from a carious temporary molar, which was removed from the mouth of a person aged 18 years. The author states, that the "pulp was found converted into a mass of crusta petrosa and dentine confounded together." The drawing is beautifully executed, and shows, by the usual indications, that the pulp-cavity has been enlarged by absorption of its parietes.

while in bone, or *cementum,* the removal of a lost portion is of frequent occurrence. Sections taken from the teeth of adults seldom fail to exhibit points where the *cementum* has been removed and again added; and very commonly the absorption has at points extended a short distance into the *dentine,* and the lost parts made good with *cementum.* This condition may be observed in perfectly sound teeth; but in unsound ones, where the *cementum* exceeds the normal amount, the removal and renewal of issue is still more marked. If the section be so made as to give a view of the surface of the pulp cavity, we shall probably find evidence of the pulp after the full development of the tooth, having resumed its full formative powers, and produced new, or secondary *dentine,* the action having been excited either by the wearing away of the tooth or by the presence of caries. If the irritation be continued until it extends down the fang as far as its extremity, and signs of inflammation show themselves, the aperture of the fang will become enlarged by absorption, and after awhile the enlargement is continued to a considerable distance up the root of the tooth. The canal may be again contracted, not by the formation of *dentine,* but by the development of *cementum;* and I have seen one or two instances in which the greater part of the pulp cavity in permanent teeth has been lined with *cementum.* This condition of tissues is very common in teeth that have been long the subject of caries, but I believe it is not confined to carious teeth. I have several specimens of temporary teeth, in which the lower part of the root has suffered from absorption, and then has become the seat of deposition of *cementum,* leaving only a small canal in the centre. High up the root small patches of *dentine* have been removed, some of which only have been made good with *cementum,* while the contiguous parts have retained their usual condition.

Judging from a view of the engraving only, it would appear that the tissue in contact with the wasted dentine is cementum only, while the newly-developed dentine is limited to the inner portion of the mass. If this view be correct, the specimen would have served for the illustration of the present paper.

It will be seen that the foregoing facts bear upon the opinions advanced by Mr. De Morgan and myself, in the paper on the structure and development of bone, before cited; that we have indications in teeth, as in bone, of alternations, of removal, and deposition of tissue. In the young subject, the development of bone tissue is in excess of absorption, allowing the bones to increase in size; that in middle life the two powers, under ordinary circumstances, balance each other, and the bones preserve their adult dimensions; while in old age the absorbent action appears to preponderate. Conditions pretty nearly parallel occur in the dental tissues after the temporary tooth has been fully formed; portions of *cementum* are removed, and with it, in some cases, a little *dentine*; the lost parts are replaced by *cementum*, and the tooth is again perfect. When the time approaches for shedding the teeth, the two actions alternate; but the absorption being in excess of the development, the tissues disappear, and the tooth is shed. After the formation of the permanent teeth we have occasional alterations of the two actions; but they are balanced, and neither increase or diminution of size is observed. But as age comes on, it often happens that absorption is in excess, the fangs diminished in size, the teeth become loose, and fall out.

*Observations on the Structure of the Enamel.*—Without going fully into the structure and development of the enamel, and into the citations of the opinions published upon the subject, I wish to take this opportunity of recording certain observations which I have made upon that structure. The transverse striation of the enamel fibres has been frequently remarked, but the cause of these markings has not been determined. If sections from a number of teeth be examined, it will be found that the striæ are much more strongly pronounced in some specimens than in others, and most especially so in those in which parts of the tissue have a brown color when seen by transmitted light.

The markings crossing the direction of the fibres are of two descriptions. The one arranged in contour lines, and situated at irregular distances from each other, uncertain in number and extent, and sometimes altogether absent. The other kind minute and regular, extending from fibre to fibre, and strongly resembling the transverse markings in voluntary muscle. In the present instance my remarks will be confined to the latter kind of markings.

In unhealthy subjects the permanent teeth, when they appear through the gums, are not unfrequently destitute of the brilliant white color common to the finely-developed organs of a healthy child; on the contrary, they have an opaque yellow color. If such teeth be selected for examination, we shall find that the sections, when reduced sufficiently thin to be seen by transmitted light, present in the enamel a confused opaque appearance; but if a tolerably high power be used (such as the quarter or eighth object-glass) in conjunction with a strong light, the dark appearance will resolve itself into a series of lines; the one set marking the course of the fibres, the other taking the direction of the transverse *striæ*. The two sets of lines crossing each other at right angles leave interspaces approaching a square form. These interspaces are filled with granular masses, having the appearance of cells. By treating the section carefully with dilute hydrochloric acid, these appearances become more distinct, and we then have series of parallel fibres composed of distinct sheaths, each containing a line of granular cells or masses arranged in a single series, presenting a strong resemblance to the ultimate fibrellæ of muscles. That such is the true structure of enamel is, I think, satisfactorily proved by specimens in my collections, some of which show the cells or granular masses; whilst others show the sheath, with the contents removed. Other specimens, again, show the enamel fibres in the very young subject, deprived of their salts, detached from each other, and floating about in the fluid in which the section is preserved.

The fibres illustrating these forms were drawn from specimens which retain the conditions figured. The appearances described do not admit of dispute; but the interpretation of their origin may perhaps be differently given by observers who do not agree upon the manner in which the enamel is developed. I do not propose to enter upon the question of development; but shall for the present leave the subject, after stating the varying conditions of enamel as it is found in human teeth.

In well-formed teeth, although the cell-like markings in the enamel are not by any means as distinct as in teeth in the condition I have described; yet having first examined the latter, but little difficulty will be experienced in recognizing here and there faint indications of a similar structure, especially if the light be well managed. The more perfect the development of the tooth, the more transparent and free from markings will be the enamel, when seen as a microscopic object; and the less perfect the more distinct will be the columns of granular cell-fibres.

Examples may readily be found in which the union between the enamel fibres is so defective that the tissue readily breaks down; a condition rendering it very difficult to grind it sufficiently thin for microscopic examination. When obtained, however, such specimens are very instructive, as they show distinctly the individual fibres and their contents, which in the most highly developed tissue are so perfectly fused together, that the strongly marked distinction of parts, which is so obvious in the one, is almost entirely lost in the other.

From what has been stated it will be seen that my view of the structure of enamel is as follows:—

The enamel fibres are composed of a sheath containing a series of cells or masses; that in perfectly developed enamel, the cells or masses and sheaths are so blended that but slight distinction of parts remains, but that in less perfectly developed tissue the component parts remain visible.

*Observations on the Development of the Enamel.*—In the preceding remarks we have referred to the structure of the enamel when fully formed. We now propose to enter upon the manner of the formation.

Mr. Huxley, in an able article published in this Journal, entered very fully into the history of the subject, giving a clear account of the different views which have been promulgated, and citing the authorities for each. Under these circumstances it will not be necessary for me to go over the same ground. I will, therefore, refer the reader to the pages which contain Mr. Huxley's paper, in place of reprinting his historical matter.*

After adopting this arrangement, that part of Mr. Huxley's paper which gives his own views on the development of the enamel, together with that which has been subsequently written upon the same subject, alone remains for consideration.

Prior to the appearance of Mr. Huxley's essay, it was pretty generally believed that the enamel fibres were formed by the direct calcification of the columns of the enamel organ. This opinion has, however, been shaken by a discovery made by that distinguished physiologist. He found that a membrane can be raised from the surface of the enamel, at any period during growth, by the addition of an acid; the membrane being external to the enamel fibres already formed, and internal to the enamel organ—in fact, lying between and separating the two tissues. This membrane Mr. Huxley regards as the *membrana preformativa* of authors. He describes it as perfectly clear and transparent, and as being continued over the dentine in those parts where enamel has not yet been formed, and over the dentinal pulp where dentine has to be developed, giving it in fact the position which the basement membrane of the

* On the Development of the Teeth, and on the Nature and Import of Nasmyth's 'Persistent Capsule;' by Thomas H. Huxley, F.R.S.—'Quarterly Journal of Microscopical Science,' No. III, 1853.

mucous membrane of the mouth would occupy when the tooth-pulp is in the follicular stage, and consequently in the sacular stage, supposing such membrane to exist in the one case, and that it has not disappeared in the other. These points are shown in the figures illustrating Mr. Huxley's paper.

M. Lent, a pupil of Kolliker's, published a paper on the development of the dental tissues, which was subjected to the Professor for revision.* Hence it must be regarded as expressing to some extent the opinions of M. Kolliker as well as those of M. Lent. The account there given of the development of the enamel is in the main but a confirmation of Mr. Huxley's statements, the points of difference being unimportant. M. Lent describes the so-called *membrana preformativa* as structureless, but as it were indented with the ends of the enamel fibres. His figure shows a surface impressed with minute square depressions. Mr. Huxley gives a similar figure. The latter author says: "Neither the capsule nor the enamel organ takes any direct share in the development of the dental tissues, all three of which—viz. enamel, dentine, and cement—are formed beneath the *membrana preformativa*, or basement membrane of the pulp." In another place he says: "Neither the capsule nor the 'enamel organ,' which consist of the epithelium of both the papilla and the capsule, contribute *directly* in any way to the development of the dental tissues, though they may indirectly.

M. Lent believes that the enamel organ exerts some direct influence in the formation of the enamel, and puts forward the following hypothesis, viz. that the cells of the enamel organ secrete a fluid, which passes through the *membrana preformativa* and there forms enamel, and he assumes that the secretions of individual cells are independent, each one forming or corresponding to an enamel fibre.

* 'Ueber die Entwicklung des Zahnbeins und des Schmelzes,' von Eduard Lent, Stud. Med. aus Hamm.' 'Zeitschrift fur Wissenchaftlichs Zoologie Sechster Band,' p. 121, 1855.

I have latterly been occupied with this subject, but have for the most part confined my investigation to young and fœtal teeth of the human subject. I must, therefore, be understood to speak of the enamel of the human teeth.

The method of investigation has been that indicated by Mr. Huxley and M. Lent; and in the pursuit of the subject I have endeavored to trace the development of the tissue without reference to its homological relations, under the belief that the structure and development of a tissue should be perfectly understood before assigning its place among other structures.

The investigations were commenced upon the lower jaw of a nine-months' fœtus, which had been in spirit for some weeks. On placing an incisor under the microscope, the surface was seen to be covered by the enamel organ: the addition of a drop of dilute hydrochloric acid (one part of acid to eleven of water) at once produced the appearance described and figured by Mr. Huxley; that is, a membrane seemed by degrees to swell up from the whole surface of the enamel, the outer surface having adherent to it, by their proximal ends, the columns of the enamel organ. The covering glass was then removed, and the acid taken up with blotting-paper, and dilute spirits of wine substituted. The next step in the investigation was the removal of the membrane raised by the acid, in order to submit it to separate examination. This end was effected by the aid of needles; but in the operation the part became torn in several places, so that its sac-like form was lost. On returning the specimen to the microscope it was seen that the membrane had a strong tendency to roll up in an opposite direction to its normal position on the tooth, the outside thereby becoming the inside of the rolls. This disposition offered facilities for examination: had it been otherwise there would have been some difficulty in obtaining a good view of the torn edge. It will be observed, on examining the figure (which is an accurate representation of a preparation which I have succeeded in preserving,) that we have on the concave side the columns of the enamel organ, while on the convex side the decalcified enamel fibres remain. I have to discover any thing like a distinct membrane interposed between the two parts. A point may be recognized where the two graduate into each other; but this part cannot be regarded as a membrane, as the forming-enamel fibres clearly pass through it.

The columns of the enamel organ are, however, very readily detached, and many float off in the fluid when the part is under manipulation. If examined in this condition, some are found in parallel bundles, and apparently attached slightly to each other; but many are quite unconnected. But whether associated or single, each column will be found to have a delicate small process projecting from that extremity which was connected with the enamel, a process which would pass through a membrana preformativa, could such be shown to exist. Immediately above the point from which the process starts, the column has, when separated from its fellows, a slight circumferential dilatation, as though the cylinder had been averted at the edge when the separation was effected. A close examination of the columns will, I think, lead to the belief that each is composed of a delicate sheath, in which is inclosed one or more nuclei, the interspaces being occupied by transparent granular matter. The nuclei are usually more distinct near the peripheral end of the columns; the attached extremity being commonly more granular than nucleated; but I have seen cases in which the sheath seemed pretty fully occupied by nuclei. After the preparation had been kept for a few weeks, the nuclei became more faint, and the granular matter more apparent.

Now, supposing the decalcified enamel fibres are detached from the columns and are viewed singly, it will be seen that the end which approached the dentine is clear and transparent, while that which meets the columns is coarse and granular, appearing by transmitted light of a deep-brown color; indeed, but for the color, it would be difficult to distinguish the distal extremity of the decalcified enamel fibre from the proximal end of the column of the enamel organ.

In many parts of the specimen the columns have been wholly detached, leaving a surface similar to that figured by Mr. Huxley, and described as the membrana preformativa. But if we look directly at the edge of the specimen where it is turned towards the observer, it will be seen that the enamel fibres pass through to the surface of this apparent membrane.

The enamel fibre, in its decalcified state, consists of a fine transparent and structureless sheath in the part which is fully formed, but in the distal portions, where development is progressing, the sheath appears to contain in many instances granular matter.

M. Lent mentions that he had at first some difficulty in obtaining the membrana preformativa, freed from enamel fibres. He at length succeeded, by treating the decalcified specimens with caustic potash or soda. No doubt the extremely delicate sheath of the enamel fibre would under such treatment soon disappear, and he might have got rid of the so-called membrane by a continued application of the same agent, in which case he might as fairly have argued that no soft tissue existed, as he has done in assuming that a distinct membrane bounds the enamel fibres because the sheaths have been dissolved by an alkali, before the partly ossified distal extremities disappeared.*

* The results of the following experiment illustrate the amount of dependence which can be placed upon membranes, the existence of which cannot be demonstrated otherwise than by the use of reagents. A thin longitudinal section was prepared from the upper incisor of a rat. This was placed for a short time in hydrochloric acid and water (one part of acid to eleven parts of water;) on removal, the acid was neutralized by a solution of potash. When placed in the field of the microscope, it was seen that membranes had started up from the whole surface of the preparation. Not only did a membrane part from the surface of the enamel, but one equally distinct peeled up from the worn, masticating surface of the tooth, while others appeared upon the surfaces which were produced in grinding the section. The membranes thus demonstrated were distinct, clear, and transparent, but exhibited no trace of the structural char-

The appearances which I have described, as existing in one specimen, may be found in the teeth of similar age in any fœtus, which has not been too long kept. Immersion in spirits of wine for a short time, I think, favors the demonstration, as the extremely-delicate columns of the enamel organ become hardened, and hence keep their normal position more frequently than in perfectly fresh subjects. Still in the latter similar structural conditions to those I have described may be observed.

If, instead of taking an incisor, the first molar of a nine-months' fœtus be selected, the tooth-sac will be found distended with a fluid, in which numerous nucleated cells float. Generally the cusps of the pulp are covered by caps of dentine, though this is not uniformly the case at this age. In several instances I have preserved specimens, in which one cusp only was invested with dentine, while the others were quite free from calcification. In the latter case the membrana preformativa should be distinctly visible. I have not been able, however, to see any thing that conveys to my mind the idea of a distinct and separable membrane. A slight amount of transparent tissue may be seen extending beyond the peripheral dentinal cells, but it also dips in between them, and has all the appearance of being nothing more than the blastema, which connects into a mass the cells of the pulp. I do not, however, propose to go into the development of dentine in the present article; hence the question of the presence or absence of a preformative membrane extending over the dentinal pulp, and the relations of such membrane to the development of dentine, may be left for future discussion.

If attention be directed to the cusps in which calcification has commenced, appearances similar to those described acters of the tissues from which they were derived, and of which they had formed a part prior to the application of the reagents. In this experiment, the action of the acid was arrested by the potash before the whole of the section had been decalcified. The edges and surfaces were softened, but the interior remained firm and retained its structural characters.

in the incisor will, if similarly treated, be found, excepting only the enamel organ, the columns of which, in this case, are shorter than those in the more advanced tooth.

Although I have confined the description to the structural conditions found in developing teeth in one jaw, my examinations have been extended over the teeth from many fœtal jaws. The results have, however, been uniformly similar.

Assuming that the foregoing observations have been correctly made, we need have no difficulty in explaining the manner in which the enamel is developed, and in accounting for the appearances exhibited in the fully-formed tissue; of which a description and figures were given in the last number of this journal. The columns of the enamel organ must be regarded as subservient to the development of the fibres, the conversion of the one into the other taking place in the following manner:—The proximal end of the column becomes calcified, not uniformly throughout its thickness, but the outer surface or sheath first receives the salts of lime, and at the same time the columns become united laterally. At this point—that is, at the extreme margin of calcification—the columns readily separate from the fibres, and leave a surface which, when looked upon directly, has the appearance of a membrane, the reticulate character of which is due to the withdrawal of the central portion of the calcifying column, this central portion being the process which has been described as forming part of the detached column. The calcification of the central part of the column goes on gradually, but does not keep pace with that of the sheath, and when calcified, presents some points of difference when compared with the surface of fibre. Thus, in adult tissue, the interior of the fibre dissolved before the surface, leaving the reticulated appearance described and figured in the last number of the Journal. Before calcification, the nuclei of the column appear to break up into subgranular matter, which may often be detected at the distal ends of the forming-enamel fibres. The situation usually occupied by well-marked oval nuclei is the distal extremities of the enamel-organ columns; but sometimes we find examples in which the nuclei, or bodies very like them, fill up the whole of the sheath, and become calcified. It may generally be found in the opaque white or brown teeth frequently seen in strumous subjects. A little practice will enable the histologist to recognize teeth which will yield specimens like the one figured.

Many authors have noticed the transverse striation of the enamel fibres. The structural condition I have described is but a more perfect development of that which is but faintly marked in the striation, and a less perfect development of the enamel itself.

In looking over a series of sections of teeth, we shall not fail to find other exceptional conditions than that I have described, and these must be also regarded as the results of imperfect development. I allude to the irregularly-granular state of enamel fibres found in patches scattered here and there amongst highly-developed tissue. At such points the granularity is in many specimens confined to the interior of the fibre, the sheath appearing clear and structureless. Indeed, this deviation from the normal state appears due to the calcification of the columns of the enamel organ, prior to that change by which the granularity disappears, and the fibre becomes transparent.

Mr. Huxley has referred to the "persistent capsule" described by Mr. Nasmyth, and considers it to be identical with the *membrana preformativa.* In several specimens which have been decalcified, after being reduced sufficiently thin for miscroscopic examination, this membrane is obviously continuous with the cementum of the fang, and in other specimens, which have not been treated with acid, I find the membrane thickened in the deep depression of the crown of molar teeth, and there tenanted by a distinct lacuna. The occurrence of these two circumstances would indicate that Nasmyth's membrane is cementum rather than membrana preformativa. The general absence of lacuna in this membrane is due to its want of sufficient thickness to contain them, just as we find these bodies wanting in the cementum of the fang when the layer of that tissue is very thin.

Apart, however, from this apparently structureless layer described by Mr. Nasmyth, we may sometimes observe a diminution in the fibrous character of the enamel at the terminations of the fibres on the surface of the tooth, and also at the terminal edge of the enamel on the neck of the tooth. In each of these situations appearances may be found which suggest the idea that a fluid blastema became calcified, and that the fibres had in the process become fused, and more or less lost in the mass so formed. Indeed, in the situation last mentioned, lamination of an indistinct character may take the place of fibres; or both the laminated and fibrous arrangement may be replaced by a structure exhibiting little arrangement of parts. In any case, however, this deviation from the normal structural character of the enamel is limited to the terminal edge of the tissue. The development of dentine and cement will form the subject of a future communication.

